# Safety of the Administration of an Inactivated PCV2a/PCV2b/*Mycoplasma Hyopneumoniae* Vaccine to Pregnant and Lactating Sows and Gilts

**DOI:** 10.3390/vaccines11091483

**Published:** 2023-09-14

**Authors:** Elena Pérez, Cristina Venegas-Vargas, Andrea Heinz, Megan Smutzer, Lucas P. Taylor, Yvette Diamondidis, Nevena Mangarova, Tara Hansen, José Angulo, Meggan Bandrick, Monica Balasch

**Affiliations:** 1 Zoetis Manufacturing & Research Spain S.L., Ctra Camprodon s/n, 17813 Vall de Bianya, Spain; elena.perez@zoetis.com; 2 Zoetis Inc., 333 Portage St, Kalamazoo, MI 49007, USA; mariacristina.venegas@zoetis.com (C.V.-V.); andrea.heinz@zoetis.com (A.H.); megan.smutzer@zoetis.com (M.S.); lucas.p.taylor@zoetis.com (L.P.T.); yvette.diamondidis@zoetis.com (Y.D.); meggan.bandrick@zoetis.com (M.B.); 3 Zoetis Belgium S.A., 20 Mercuriusstraat, 1930 Zaventem, Belgium; nevena.mangarova@zoetis.com; 4 Zoetis Inc., 601 West Cornhusker Hwy, Lincoln, NE 68521, USA; tara.hansen@zoetis.com; 5 Zoetis Inc., Parsippany, NJ 07054, USA; jose.angulo@zoetis.com

**Keywords:** porcine circovirus diseases, porcine circovirus vaccines, gilt development

## Abstract

Porcine Circovirus type 2 (PCV2) vaccination of gilts during acclimation has become a routine practice in commercial pig farms to homogenize herd immunity to PCV2 and reduce the impact of diseases associated with PCV2 infection, namely reproductive, respiratory, systemic, and other PCV2-associated diseases. The periodic mass vaccination of sows, with the same objectives, is also common. To ensure mass vaccination is an appropriate health management tool, demonstrating that the vaccine is safe in different sow/gilt physiological stages is necessary. The objective of the present studies was to evaluate safety of a PCV2a/PCV2b/*Mycoplasma hyopneumoniae* (PCV2a2bMHP) killed vaccine in sows and gilts during gestation and lactation, under controlled experimental pen conditions, and during gestation, mimicking mass vaccination, under field conditions. Safety was assessed by monitoring for immediate adverse reactions after vaccination, rectal temperatures after vaccination (controlled experimental pen studies only), local and systemic reactions, and reproductive performance (studies conducted during pregnancy) or lactation performance (studies conducted during lactation). In total, 416 sows/gilts were enrolled, and more than 4000 piglets were observed during their first week of life, under field conditions. In both controlled experimental and field studies, no immediate anaphylactic type reactions were observed after vaccination and the incidence of adverse events, such as depression or decreased appetite, was acceptable for what is expected in a swine herd. In the studies conducted during gestation, vaccination did not significantly increase rectal temperature of the vaccinated animals. Sow reproductive outcomes were not affected by vaccination. The farrowing rate of animals participating in the field study was higher than the historic averages of the farms. In the laboratory studies conducted during the first and second half of gestation, no differences in reproductive outcome were observed between vaccinated and non-vaccinated animals. However, sows vaccinated during lactation experienced a transient hyperthermia which did not affect milk production since the piglets’ average daily weight gain was not affected. The previously described results confirm that the administration of a PCV2a2bMHP vaccine was safe in the tested conditions. All the anticipated benefits of sow and gilt PCV2 vaccination, such as homogenization of PCV2 antibody titers or reduction in PCV2 circulation in the herd, would not be masked by potential adverse events due to herd vaccination. In conclusion, the administration of a PCV2a2bMHP vaccine to sows and gilts during different stages of gestation and during lactation is safe.

## 1. Introduction

Although Porcine Circovirus type 2 (PCV2) was first characterized in 1998 associated with a novel pig disease first called Postweaning Multisystemic Wasting Syndrome (PMWS) [1]), retrospective studies have been able to detect it in pig tissues since 1962 and fulfilling disease criteria in sick pigs since 1985 [2].

The disease described in the first reported cases of PMWS was characterized by wasting (weight loss, emaciation), respiratory disease (dyspnea, tachypnea), and pallor/jaundice, and less frequently, diarrhea and abnormal central nervous system clinical signs [3]. However, in the years following these first descriptions, PCV2 was systematically associated with several other pathologies. Therefore, the terms Porcine Circovirus Diseases (PCVD) or Porcine Circovirus Associated Diseases (PCVAD) were proposed to represent the group of all these conditions [4]. Due to the multiple pathologies being associated with the infection of pigs with PCV2, a nomenclature was proposed for these conditions: PCV2-systemic disease (PCV2-SD, to replace PMWS), PCV2-subclinical infection (PCV2-SI), PCV2-reproductive disease (PCV2-RD), and porcine dermatitis and nephropathy syndrome (PDNS) [5].

The disease impacts the growth and body weight of affected animals, with variable morbidity and mortality, but reaching 60% and 20%, respectively, in severe cases [6]. Therefore, a severe economic impact was foreseen in different kinds of pig herds, prior to the implementation of vaccination programs, and was economically quantified [7]. At the farm level, the greatest proportion of negative economic impact was attributed to PCV2 subclinically infected pigs. The economic impact for the English pig industry for the year 2008, prior to the introduction of PCV2 vaccines, was estimated at GBP 52.6 million per year, and approximately GBP 88 million per year during the epidemic period [7]. 

The introduction of PCV2 inactivated vaccines completely changed the scenario worldwide. The application of PCV2 vaccines represented by far the best system to control the disease under farm conditions. Moreover, vaccination of subclinically infected pigs demonstrated a significant improvement in average daily weight gain and it was the most cost-efficient strategy to reduce the economic impact of virus infection [8,9].

Several commercial PCV2 vaccines, alone or in combination with other antigens, are available worldwide, although the number may be different depending on the country. All of them are based on whole inactivated viruses or are subunit based and are intended either for sows and gilts and/or piglets. Those intended for the breeding herd confer passive immunity to piglets through colostrum, while those intended for piglets induce the development of active immunity in the target animals [9].

The success of piglet vaccination relies on the age of vaccination and/or the non-interference of maternal-derived immunity with vaccination. Although it has been described that piglet vaccination (with vaccines available at/before 2021) is effective with respect to production variables and reducing viremia, even in the presence of high maternal derived antibodies, an age of 3 weeks at vaccination is most beneficial [10]. Notably, most of the vaccines in the market have maternal immunity interference warnings on their label. It has been described that sow vaccination prior to mating elicits a strong, homogeneous humoral response in sows and, in consequence, more homogeneous colostral anti-PCV2 antibody concentrations across sows [11]. While the main licensed use of PCV2 vaccine directed to sows and gilts is to offer passive immunity to the offspring and protect piglets from PCVD, there are additional benefits of sow/gilt vaccination [9,12,13]. The use of PCV2 vaccines in sows and gilts leads to a reduction in circulation of PCV2 within the breeding herd, and in consequence, reduces the circulation of PCV2 in newborn piglets [14]. Additionally, sow vaccination has been shown to have a positive effect on reproductive variables, increasing the prolificacy and virality of piglets [15,16]. 

Considering the benefits that gilt/sow vaccination can bring to the breeding herd, e.g., reduction in PCV2 circulation and homogenizing the herd immunity, it is key to demonstrate that PCV2 vaccines are safe when used in different stages of gestation and during lactation. The objective of the present studies was to evaluate the safety of a PCV2a/PCV2b/*Mycoplasma hyopneumoniae* (PCV2a2bMHP) killed vaccine in sows and gilts during gestation and lactation, under controlled experimental conditions, and during gestation, mimicking mass vaccination, under field conditions.

## 2. Materials and Methods

### 2.1. Study Design

Two controlled experimental pen studies and one field study were conducted in pregnant sows and gilts; one laboratory study was conducted in lactating sows. 

In the controlled experimental pen studies (Table 1), 2 mL of a PCV2a2bMHP vaccine batch with all antigens at maximum potency was administered intramuscularly in the neck muscle twice at a two-week interval to gilts and sows during the first half of gestation (before 57 days of gestation), the second half of gestation (after 57 days of gestation), or during lactation. This dosing regimen represented a repeated administration of a single dose. These studies were randomized, blinded, and negatively controlled, and were performed with gilts and sows that tested negative for PCV2 genome and had negative to low antibody titers against PCV2 and MHP. All sows and gilts were monitored for immediate adverse reactions after vaccination, rectal temperatures during 4 days after vaccination, local and systemic reactions 14 days after vaccination, and reproductive performance (studies conducted during pregnancy) or lactation performance (studies conducted during lactation).

In the field study (Table 2), 2 mL of a PCV2a2bMHP vaccine batch with all antigens at medium potency (release level) was administered intramuscularly, once to gilts and sows during the first, second, and third stage of gestation. This study was randomized, blinded, and negatively controlled, and was performed with gilts and sows belonging to two different high-health farms in the USA. The virological and serological PCV2 status was not tested. All sows and gilts were monitored for local and systemic reaction within 4 h of vaccination and the following day. From Day 2 until one-week post-farrowing, all sows and gilts were observed, and any adverse health events were recorded; however, after 14 days adverse health events were recorded only if required veterinary intervention and/or treatment was required. Reproductive performance was monitored, as well as piglet health status for 1 week after birth.

### 2.2. Animals

In the controlled experimental pen studies, cross-bred gilts from a high-health farm were used. The gilts were 8–15 months of age at first vaccination and were seronegative or low seropositive to PCV2 and *Mycoplasma hyopneumoniae* (MHP). None of the gilts were PCV2 viremic at first vaccination. Gilts were housed in gestation pens and in farrowing crates. Cross fostering in the lactation study was allowed only before Day 0.

In the field study, sows and gilts from two commercial farms in the USA were enrolled. The herds were of high health status. No clinical signs of PCV2 or MHP were present in the herds at the time of vaccination. All parities were represented at both sites. Sows/gilts were housed in conventional gestation crates (Site A) or gestation pens (Site B) and in farrowing crates for both sites. The piglets were kept with their birth sow throughout the study when possible. Cross fostering was permitted for animal welfare reasons only.

Farm A was a 1300-sow farrow-to-wean closed/internal multiplication facility, while farm B was a 600-sow farrow-to-wean facility. Both sites vaccinated gilts/sows with a Rotavirus, *Clostridium perfringens* Type C and *Escherichia coli* bacterin toxoid two weeks prior to their anticipated farrowing dates. Average production variables are shown in Table 3.

### 2.3. Vaccination

The PCV2a2bMHP vaccines used in the controlled experimental and field studies were similar to CircoMax Myco^®^ and Fostera Gold^®^ PCV MH, respectively. The composition of these vaccines is the same and both are produced by the same manufacturer (Zoetis Inc, Parsippany, NJ, USA). The vaccine consists of a recombinant PCV2a and PCV2b product based on two killed whole chimeric viruses containing the genomic backbone of the non-pathogenic porcine circovirus type 1 but replacing its ORF2 capsid gene by that of PCV2a or PCV2b. Moreover, it contains a MHP bacterin, and 10% SP Oil adjuvant (oil in water emulsion).

For the objective of these studies, the potencies of the antigens used to manufacture the respective batches were at maximum release potency for the controlled experimental pen studies and at commercial potency for the field study. 

On study Day 0, the assigned treatment, according to the random treatment assignment plan, was administered by a dispenser. Only healthy animals, as determined during the clinical observations prior to vaccination on Day 0, were vaccinated. Two mL of each test material was administered intramuscularly with a 1.1 × 40 mm needle/2 mL syringe, in the right neck side (Day 0) and left neck side (Day 14) (laboratory study), or in the right neck side only (Day 0) (field study) with a 14–16 G or 18 G, 1″–1 ½″ needle.

In the controlled experimental pen studies, randomization was performed with an SAS program specific for the study using a random number generator function to generate all random numbers in the program. Treatments were randomly assigned to animals using a generalized randomized block design. In the study conducted during lactation, blocking was based on the room; in the studies conducted during gestation, blocking was based on PCV2 serology. Treatments were balanced as close as possible within each room.

In the field study, within each site and gestation period, treatments were randomized to pigs using a generalized randomized block design using a SAS (Cary, NC, USA) computer program and a random number generator function. The random number generator was assigned a random number for each order of enrollment. Blocking was based on order of enrollment in a 2:5:5 ratio. For each group of 12 orders of enrollment, a random number was sorted in ascending order. The lowest 2 random numbers were assigned to T01, the next 5 random numbers were T02, and the highest 5 random numbers were T03.

### 2.4. Clinical Observations

In the controlled experimental pen studies, clinical signs were monitored for individual animals once on Day −1, twice on Day 0 and Day 14 (prior to vaccination and 3–6 h post-vaccination), and once daily from Day 1 through Day 13 and from Day 15 through Day 28. Moreover, gilts were observed immediately after each vaccination to record any potential systemic reaction (e.g., anaphylactic type reaction). Rectal temperatures (RT) were obtained once prior to each vaccination day on Day −1 and Day 13 (as baseline), twice on Day 0 and Day 14 (prior to vaccination and 3–6 h post-vaccination), and once daily for 4 consecutive days (from Day 1 through Day 4 and from Day 15 through Day 18). A calibrated digital thermometer was used. Pyrexia was defined as RT ≥ 40.5 °C.

In the field study, individual animal observations were performed after vaccination the day of vaccination (within 4 h of vaccination) and the day following vaccination. On Days 2–14, animals were observed according to site husbandry practices and all abnormal observations were recorded. On Day 15 until one week of post farrowing, all animals were observed and only animals with an adverse event (AE) that required veterinary intervention or treatment were recorded. 

### 2.5. Injection Site Observations

In the controlled experimental pen studies, a qualified person observed and palpated injection sites for local reactions. Local reactions at the injection site were evaluated once on Day −1 and Day 13 (as baseline, prior to each respective vaccination), twice on Day 0 and Day 14 (prior to vaccination and 3–6 h post-vaccination), and once daily for 14 days after each vaccination. In other words, injection site observations were collected on Days 1 through 13 (on the right-neck side) and from Days 15 through 28 (on the left-neck side). 

In the field study, injection sites were observed on Day 0 and 1. Since there were no injection site reactions observed on Day 1, further observations were not necessary.

### 2.6. Reproductive Outcomes and Litter Health Observations

In the controlled experimental pen studies conducted with gestating gilts, all animals were followed until farrowing, when reproductive performance was individually registered: date of birth and total born piglets (born alive, stillborn, or mummified).

In the field study, farrowing data for sows/gilts were collected within 24 h of farrowing and included number of piglets born alive normal, stillborn, mummified, and with low viability. Live born piglets from the sows or gilts were observed according to site husbandry practices from the day of birth until piglets were one week of age. All abnormal clinical signs observed were recorded.

### 2.7. Lactating Performance

In the controlled experimental pen study conducted with lactating sows, the piglet body weight was used as an estimate of lactation performance. Piglets were weighed on Days 0, 7, 14, and 21 (until weaning) to calculate the average daily weight gain (ADG) for each treatment group.

### 2.8. Data Analysis

In the controlled experimental pen studies, frequency distributions of whether or not an animal had a systemic reaction were calculated for each treatment and time point. It was determined if an animal ever had a systemic reaction following each vaccination and overall. Frequency distributions of ever having a systemic reaction were calculated for each treatment and vaccination as well as overall. 

For rectal temperatures, the model included the fixed effects of treatment, time point, and treatment by time point interaction and the random effects (which varied from study to study due to housing). Pair-wise treatment comparisons were made at each time point if the treatment or treatment by time point interaction effect was significant (*p* ≤ 0.05). Treatment least squares means, 95% confidence intervals, and the minimum and maximum were calculated for each time point.

Frequency distributions of reaction scores, redness, pain at palpation, local temperature at palpation, and swelling were calculated for each treatment at each time point of data that was collected. In addition, frequency distributions of whether or not an animal had each of the above injection site reactions (if score = 0, then No, if score ≠ 0, then Yes) was calculated for each treatment by vaccination and overall.

Duration of injection site reactions was calculated for each animal and vaccination (1st, 2nd and overall). Descriptive statistics, means, standard deviations, and ranges were calculated for each treatment and vaccination (1st, 2nd and overall).

The percentage of piglets from each litter that are live births, normal (live piglets minus low viability), stillborn, mummies, and have low viability was calculated and transformed with an arc sin square root transformation and analyzed with a general linear mixed-model analysis. The model included the fixed effect of treatment and the random effects of room and block within room. Pair-wise treatment comparisons were made if the treatment effect was significant (*p* ≤ 0.05). Treatment back-transformed least squares means, 95% confidence intervals, and the minimum and maximum were calculated.

Piglet body weight was analyzed using a general linear repeated measures mixed-model analysis. The model included the fixed effects of treatment, time point, and treatment by time point interaction and the random effects of block, sow within block by treatment, piglet within sow, and sow within block, treatment, and time point. Pair-wise treatment comparisons were made at each time point if the treatment or treatment by time point interaction effect was significant (*p* ≤ 0.05). Treatment least squares means, 95% confidence intervals, and the minimum and maximum were calculated for each time point. Additionally, linear functions of the least squares means for body weights were used to calculate estimates of the average daily gain for each period. Average daily gain treatment comparisons were also made at each period if the treatment effect or treatment by time point interaction was significant.

In the field study, data were summarized by treatment, by site and treatment, and by site, gestation, and treatment. Additionally, serials were combined as vaccinates (T02 and T03) and controls (T01) for all summaries. For litters health observations and farrowing outcome, data were summarized by combined treatment and stage of gestation.

It was determined if an animal ever had an AE (different adverse events were tabulated separately). Frequency distributions were calculated for whether a pig ever had an AE. It was also determined if an animal ever had an AE (different AE were tabulated separately) that was attributable to the IVP. Frequency distributions were calculated for whether a pig ever had an AE attributable to the IVP.

The percentage of the litter that was abnormal for each abnormal health event (abnormal, dead/euthanized, diarrhea, abnormal breathing, lameness, runt, congenital musculo-skeletal, and other) was calculated. The percentage of the litter was transformed with an arc sin square root transformation and was summarized with descriptive statistics, back-transformed means, standard deviations, and ranges for each treatment and time point. The cumulative percent that died/euthanized was calculated for each treatment. Frequency tables for the reason for the found dead/euthanasia of piglets were calculated for each treatment.

Frequency of all injection site reaction scores were calculated for each treatment and time point data were collected.

Live births (normal + low viability), normal, low viability, stillborn, and mummies were calculated. The percentage of the litter that was live, normal, stillborn, mummified, and had low viability was transformed using an arc sin square root transformation and summarized with descriptive statistics, means, standard deviations, and ranges, and calculated for each treatment. The results were back-transformed for presentation.

Frequency tables of sow outcome were calculated for each treatment.

## 3. Results

### 3.1. Controlled Experimental Pen Studies

No systemic reactions were observed immediately after the administration of the vaccine in any of the treatment groups. 

Regarding clinical signs during the experimental period, no abnormal clinical observations were found in the study performed during the first half of gestation. 

In the study conducted during the second half of gestation, two adverse events were recorded. One sow aborted at study day 31 (17 days after the second vaccination). A complete diagnostic abortion panel to rule out an infectious etiology was conducted, and the baseline reproductive data for the farm of origin were also checked. PCR diagnostic of Porcine Reproductive Respiratory Syndrome Virus (PRRSV), Porcine Parvovirus (PPV), Leptospira, *Chlamydiaceae*, PCV2 and PCV3 were performed from foetal heart (PCV2 and PCV3 PCR), lungs (PPV PCR), thymus and tongue (PRRS PCR), eye fluid, liver and kidney (*Lesptospira* LIPL-32 PCR), and gastric fluid (*Chlamydiaceae* PCR). Blood was collected from the sow that aborted 9 days after the abortion on Study Day 40 to perform the following laboratory analyses on serum samples: PPV indirect haemagglutination, Swine Influenza virus ELISA, Swine Erysipelas serology, and PRRSV ELISA. Direct detection or seroconversion to the listed pathogens were not achieved with any of the samples.

Another sow was found dead at study Day 44, 30 days after the second vaccination. At necropsy, a uterus torsion was diagnosed.

In the study conducted during lactation, one sow was found dead at study Day 1 (1 day after 1st vaccination). At necropsy, a gastric rupture was diagnosed upon necropsy.

Regarding rectal temperatures (Table 4), no significant differences between vaccinated and non-vaccinated groups were observed in the studies conducted during the first and second halves of gestation. In contrast, in the study conducted during lactation, vaccinated gilts had a significantly higher (*p*-value ≤ 0.05) rectal temperature than control gilts on study days 15 and 17 (1 and 3 days after second dose).

Injection site reactions were not detected in the study conducted during the first half of gestation nor after the first vaccination of the studies conducted during second half of gestation and during lactation. However, after the second vaccination in the study conducted during the second half of gestation, 70% of the sows (7 out of 10) had a very mild swelling (< 0.5 cm in diameter) for 1 day after the second vaccination, except one sow which had a moderate swelling (0.5–2 cm) that lasted for 11 days. After the second vaccination in the study conducted during lactation, 12.5% of the sows (1 out of 8) had a very mild swelling (less than 0.5 cm in diameter) of 2 days of duration. No reactions were detected in the control sows.

In the studies conducted during the first and second half of gestation, no statistical differences in the percentage of alive, normal, low viability, mummified, or stillborn piglets were observed between vaccinated and non-vaccinated animals (Table 5).

In the study conducted during lactation, no significant differences in piglet body weight or average daily weight gain (ADWG) were observed between vaccinated and non-vaccinated animals (Table 6 and Table 7).

### 3.2. Field Study

Adverse events were recorded as described in the two farms (Table 8). None of the adverse events were attributable to the vaccine as determined by the study investigators.

Injection sites were observed on Day 0 and 1. Since there were no injection site reactions observed on Day 1, further observations were not necessary.

Reproductive sow outcomes were recorded (Table 9). All Site A animals (historic conception and farrowing rates of 85% and 84%, see Table 3) farrowed with the exception of one animal which was found dead. This death was determined to not be related to the vaccine by the Investigator. All Site B animals farrowed with the exception of three animals that aborted, ten animals that were not pregnant (upon pregnancy recheck via ultrasound post vaccination), and two sows that were culled. The historic conception rate for Site B was 69% and the farrowing rate was 60.7%. In summary, 0.7% of the vaccinated sows and 1.7% of the controls experienced an abortion; the site’s historic abortion rate was 2%. The abortions were determined by the investigator as unknown, but unlikely related to the vaccine and within the standard ranges of the farm.

Farrowing outcomes (born alive, born normal, low viability, stillborn, and mummies) were calculated based on the number of litters, number of piglets per litter, and number of piglets per litter fitting each variable (Table 10). As the litters have a different number of piglets, the denominator is specific by litter.

The vaccinated group had numerically higher percentages of piglets per litter born alive, born normal, and mummies compared to controls. The vaccinated group had 7.1% and 1.1% of stillborn and low viability piglets per litter, respectively, while the control group had 10.5% and 1.1%, respectively.

**Figure 1:**
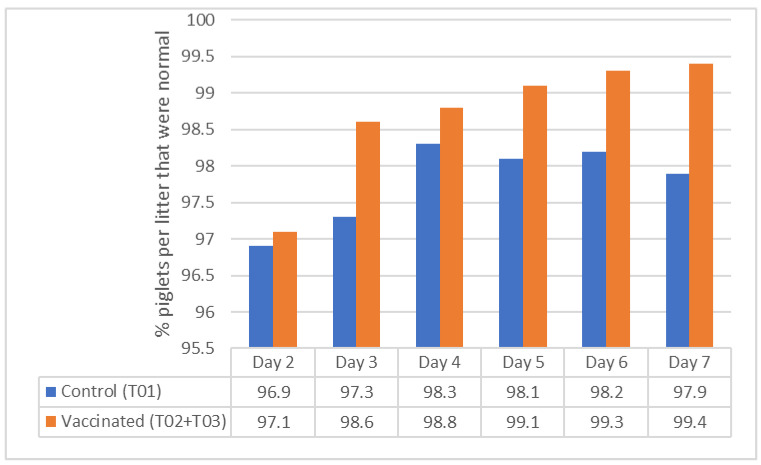
Back-transformed mean percentage of piglets per litter that were normal (field study).

Litter health observations (normal, abnormal, found dead, euthanized, diarrhea, abnormal breathing, lameness, runt, congenital musculoskeletal, and other) were calculated based on the number of litters on each day (Day 1 to Day 7), number of piglets per litter per each day, and number of piglets per litter fitting each variable on each day. As the litters have different numbers of piglets, the denominator is specific to litter on each day. Day 1 started 24 h after the sow farrowed. Greater than 90% of piglets per litter were normal for each group on each day (Figure 1). At both sites, the mean percentage of piglets per litter observed with the following clinical signs was zero: abnormal breathing, diarrhea, lameness, congenital musculo-skeletal disorder, and others.

## 4. Discussion

Sow and gilt vaccination with PCV2 vaccines is becoming a standard practice since it elicits a strong, homogeneous humoral and cellular immune response and, in consequence, more homogeneous colostral PCV2 antibody concentrations [11]. In addition, this practice has shown an improvement in reproductive performance [13]. Gilt acclimation programs frequently include PCV2 vaccination; for instance, more than 60% of the swine herds in Belgium reported PCV2 vaccination during acclimation, even if not all the PCV2 vaccines available in the market are authorized for this use [17]. The objective of the current studies was to evaluate safety of a PCV2a/PCV2b/*Mycoplasma hyopneumoniae* (PCV2a2bMHP) killed vaccine in sows and gilts during gestation and lactation, under controlled experimental conditions, and during gestation, mimicking mass vaccination, under field conditions.

Clinically, sow and gilt vaccination was demonstrated to be safe under all tested conditions (controlled and field conditions) and in all physiological stages (pregnant and lactation). No immediate anaphylactic type reactions were observed and the incidence rate of events was appropriate for what is expected in a swine herd. Further, all abnormal events were not biologically or clinically relevant as the control and vaccinated groups experienced very similar rates. Adverse reactions described either in the controlled experimental studies (incidental uterus torsion, gastric rupture, or spontaneous abortion) or in the field study (depression or decreased appetite in similar percentages than those described for the non-vaccinated sows) had an obvious root cause and were not considered to be associated with the vaccine.

A careful investigation was conducted on the abortions that occurred in two of the studies. In the abortion which occurred after vaccination during the second half of gestation, no infectious involvement was found in foetal samples tested, nor did sows seroconvert to the most frequent pathogens involved in abortion. The spontaneous abortion incidence in the farm of origin was investigated; in the last two years, spontaneous abortions accounted for 1.41 to 3.80% of pregnancy loss, which are similar rates to the 1.82% that occurred in the lab safety study (this reflects the 1 abortion). Therefore, it was reasonably concluded that the abortion percentage detected in these studies was similar to the ones recorded in the farm of origin and, in consequence, the abortion detected in this study might be considered a spontaneous abortion and unlikely related to the vaccination. Considering the period of the year when the abortion occurred (early December), the condition of seasonal abortion should also be taken into account as a potentially sound explanation [18].

In the field study, 0.7% of the vaccinated sows and 1.7% of the controls experienced an abortion, respectively; the site’s historic abortion rate was 2%, providing evidence that the vaccine did not influence the abortion rate.

Rectal temperatures after vaccination were monitored in the controlled experimental studies. In those conducted during gestation, vaccination did not impact rectal temperatures since no significant differences between vaccinated and non-vaccinated gilts were observed. However, sows vaccinated during lactation experienced a transient increase in rectal temperatures at days 1 and 3 after the second vaccination. It is noteworthy that the mean rectal temperature was not indicative of hyperthermia (39.0 °C). The maximum rectal temperature detected in the control sows was 40.3 °C, and in the vaccinated sows was 40.7 °C. One vaccinated sow was pyrectic (40.7 °C) during the 3 days after the second vaccination and returned to normal at Day 4. Heat has been demonstrated to affect milk production in sows [19]; one might think that hyperthermia might have affected milk production. However, analysis of piglet body weight demonstrated that no differences in ADWG occurred since values of both groups were not significantly different in all time periods considered. The mean body weight of the piglets from the pyrectic sow was higher than the mean of the whole group, suggesting no impact of hyperthermia in piglet body weight and, in turn, in sow milk production. A normal increase of 1.0 °C in rectal temperature can occur starting 24 h before farrowing and remain elevated until weaning, reaching as high as 40.5 °C [20]. In this case, the hyperthermia observed in two sows during lactation could still be physiological.

Based on the field study and the large number of piglets (~4500+) observed during the first week of life, there was no indication that the vaccination had an impact on the growth and development of the piglets. If milk yields had been decreased or impacted, the piglets’ health would have been impacted.

Reproductive sow outcomes did not point to any effects due to sow vaccination. The farrowing rate of animals participating in the field study was greater than the historic site averages of the farms.

Stillborn piglets accounted for 10.5% in the control litters and 7.1% in the vaccinated litters. According to the benchmarking parameters provided by Stalder (2017), the average number of total born piglets per litter in the USA was 13.9. Further, according to the same report, the industry benchmark for stillborn and mummified piglets was 1.37 +/− 0.57 piglets per litter. Calculating for a litter size of 13.9 and applying the study results of 10.5% and 7.1% stillborn for the control and vaccinated groups, respectively:

Control group = 1.5 stillborn in a litter of 13.9 piglets (13.9 × 10.5% = 1.5 piglets)

Vaccinated group = 1 stillborn in a litter of 13.9 piglets (13.9 × 7.1% = 1 piglets)

Study values for stillborn piglets per control (1.5) and vaccinated (1) litters are in line with industry standard parameters for stillborn and mummified piglets (1.37 +/− 0.57). In accordance, study values for mummies and those born alive were also within the industry standards [21]. Therefore, farrowing outcomes were not adversely affected by the vaccine or control product.

The benefits of sow/gilt PCV2 vaccination are widely accepted and as such PCV2 vaccination is becoming a widespread practice in swine herds [17]. Benefits can be as wide as providing passive immunity to neonatal pigs [12,13], reduction of PCV2 circulation within neonatal units [14], and helping with the homogenization of piglet antibody titers to PCV2 [11].

A homogeneous immunity in piglets, avoiding litters with high antibody titers, and litters with lower antibody titers helps to avoid subpopulations of weaned piglets with different immune status. Development of cellular immunity has been demonstrated for several PCV2 vaccines in the market [22,23], which induce a PCV2-specific IFN-ɣ response after one or two doses of vaccine [23]. Maintaining homogeneous immunity is key to a successful vaccination: applying the vaccine during the correct vaccination window (the period in which maternal immunity is low enough to allow development of active immunity without interfering with the immune response to vaccination) is key for the induction of active immunity. It is reasonable to assume finding the right vaccination window would be easier when the herd immunity status is homogeneous.

With all these potential benefits, the demonstration of the safety of the administration of the PCV2a2bMHP-inactivated vaccine to sows and gilts in different physiological stages is key to progress with the control of PCVD in swine herds.

## 5. Conclusions

The administration of a PCV2a2bMHP-inactivated vaccine to sows and gilts during different stages of gestation, under controlled experimental and field conditions, and during lactation under controlled experimental conditions was demonstrated to be safe. Vaccinated sows and gilts did not show signs of systemic reactions and experienced no impact on farrowing outcome or reproductive performance. Further, vaccination did not impact lactation performance as indirectly measured by piglet health and body weight.

## Figures and Tables

**Table 1:** Experimental design, controlled experimental pen studies.

Study	Treatment	N Sows/Gilts	Vaccination Schedule	End of Study
First half of gestation	Saline 2 mL	10	First dose (D0): D30–35 of gestation Second dose (D14): D44–49 of gestation	At farrowing
	PCV2a2bMHP 2 mL	10		
Second half of gestation	Saline 2 mL	9	First dose (D0): D69–71 of gestation Second dose (D14): D83–85 of gestation	At farrowing
	PCV2a2bMHP 2 mL	10		
Lactation	Saline 2 mL	9	First dose (D0): D6–15 after farrowing Second dose (D14): D20–29 after farrowing	At weaning
	PCV2a2bMHP 2 mL	9		

**Table 2:** Experimental design, field study.

Group	Treatment	Gestation Day	Dose/Route	N	Vaccination Timing	End of Study
T01	Saline or water	23–34	2 mL/IM	20	Day 0	1 week after farrowing
		61–75		20		
		79–89		20		
T02	PCV2a2bMHP Batch A	23–34	2 mL/IM	49	Day 0	1 week after farrowing
		61–75		50		
		79–89		50		
T03	PCV2a2bMHP Batch B	23–34	2 mL/IM	50	Day 0	1 week after farrowing
		61–75		49		
		79–89		51		

**Table 3:** Historical average reproduction performance parameters in farms A and B of the USA field study.

	Farm A	Farm B
Conception rate	85%	69%
Farrowing rate	84%	60.7%
Abortion rate	<1%	2%
N° piglets born/litter	15	8.85
N° piglets weaned/litter	12	8.10
Stillborn/litter	9.5%	11.8%
Mummies/litter	3%	2.1%

**Table 4:** Analysis of rectal temperatures among treatment groups within days.

		Day −1	Day 00	Day 00 Time 2 ^3^	Day 01	Day 02	Day 03	Day 04	Day 13	Day 14	Day 14 Time 2	Day 15	Day 16	Day 17	Day 18
First half of gestation	T01	38.00	37.70	37.90	37.60	38.00	37.80	37.40	37.80	37.90	37.80	37.50	37.80	37.90	37.80
	T02	38.30	38.00	38.10	37.70	37.90	37.80	37.80	38.00	37.90	37.60	37.80	37.70	37.60	37.80
	*p*-value ^1,2^	0.1255	0.1475	0.1967	0.7587	0.5554	0.8649	0.0319	0.1932	0.7379	0.4480	0.1129	0.7001	0.0333	0.6970
Second half of gestation	T01	37.80	37.00	37.80	37.60	37.50	38.20	37.90	37.80	37.60	38.00	37.50	37.60	38.00	37.00
	T02	38.10	37.40	38.10	37.60	37.40	37.80	37.80	37.80	37.70	37.90	37.30	37.30	38.00	37.00
	*p*-value ^1,2^	0.1619	0.0692	0.2562	0.9768	0.9383	0.0959	0.5245	0.9595	0.6956	0.6381	0.3641	0.0812	0.9680	0.9468
Lactation	T01	38.40	38.40	38.90	38.50	38.50	38.70	38.10	38.50	38.10	39.00	37.80	38.00	38.00	38.50
	T02	38.40	38.40	39.10	38.80	38.50	38.50	38.30	38.30	38.50	39.30	39.00	38.70	38.80	38.20
	*p*-value ^1,2^	0.7219	0.7647	0.5529	0.2339	0.9069	0.4258	0.1960	0.5119	0.2033	0.2140	0.0066 *	0.1193	0.0282 *	0.3514

^1^ When the overall F-test for treatment and the treatment by time interaction are >0.05, their corresponding contrasts are not examined and are declared not significant; ^2^ *p*-values > 0.05 are designated as not significant and *p*-values are ≤0.05 are designated as * (significant); ^3^ time 2 = 3–6 h post-vaccination.

**Table 5:** Significance values for a priori contrasts among least squares means of percentage of piglets born alive, with low viability, mummified, normal, and stillborn.

		Alive	Low Viability	Mummified	Normal	Stillborn
First half of gestation	T01	95.1	0.4	0.1	93	4.1
	T02	96.2	0.3	0.1	94.5	2.7
	*p*-value ^1^	0.728	0.9217	0.8857	0.6749	0.6193
Second half of gestation	T01	85.3	0.6	3.1	83	8.5
	T02	94.9	0.2	0.2	94.1	4.2
	*p*-value ^1^	0.153	0.4948	0.1198	0.1161	0.3739

^1^ When the overall F-test for treatment and the treatment by time interaction are >0.05 their corresponding contrasts are not examined and are declared not significant; *p*-values > 0.05 are designated as not significant.

**Table 6:** Piglet body weight (kg) least squares means, standard errors, and ranges by treatment and time point.

	Time Point	Nº obs ^1^	LSM ^2^	SE ^3^	Lower 95% CL ^4^	Upper 95% CL	Range Weight
T01	Day 00	98	2.8	0.27	2.0	3.6	1.55 to 4.22
	Day 07	98	4.2	0.28	3.4	4.9	1.81 to 6.11
	Day 14	95	5.5	0.29	4.8	6.3	1.81 to 7.9
	Day 21	96	7.1	0.30	6.4	7.8	2.79 to 10.03
T02	Day 00	92	2.6	0.24	2.0	3.2	1.43 to 5.12
	Day 07	82	4.0	0.24	3.4	4.6	1.99 to 5.52
	Day 14	82	5.3	0.25	4.7	5.9	2.54 to 6.52
	Day 21	82	6.8	0.26	6.2	7.4	3.32 to 8.63

^1^ obs: observations; ^2^ LSM: least squares means; ^3^ SE: standard error; ^4^ CL: confidence limit.

**Table 7:** Estimates of average daily weight gain (ADWG) and comparisons of ADWG treatments.

	ADWG	SE ^1^	Difference in ADWG	SE of Diff. in ADWG	2-Tailed *p*-Value ^2^	Significance of 2-Tailed *p*-Value ^3^
0 to 7 ADWG/T01	0.17	0.01	0.00	0.01	Not tested	NS
0 to 7 ADWG/T02	0.17	0.01				
0 to 14 ADWG/T01	0.18	0.01	0.00	0.01	Not tested	NS
0 to 14 ADWG/T02	0.18	0.00				
0 to 21 ADWG/T01	0.20	0.01	−0.01	0.01	Not tested	NS
0 to 21 ADWG/T02	0.19	0.00				
7 to 14 ADWG/T01	0.17	0.01	−0.01	0.01	Not tested	NS
7 to 14 ADWG/T02	0.16	0.01				
7 to 21 ADWG/T01	0.20	0.01	−0.01	0.01	Not tested	NS
7 to 21 ADWG/T02	0.19	0.00				
14 to 21 ADWG/T01	0.20	0.01	−0.01	0.01	Not tested	NS
14 to 21 ADWG/T02	0.19	0.01				

^1^ SE: standard error; ^2^ when the overall f-test for the treatment and the treatment by time interaction is >0.05, their corresponding contrasts are not examined and are declared not significant; ^3^ *p*-values >0.05 are designated as “NS” (not significant).

**Table 8:** Frequency distribution of sow adverse health events by treatment; number and percentages in brackets.

	Control (T01)	Vaccinated (T02 + T03)
Total number of animals (percentage)	60 (100%)	299 (100%)
Normal	47 (78.3%)	268 (89.6%)
Cough	1 (1.7%)	0 (0%)
Decreased appetite	2 (3.3%)	13 (4.3%)
Depression	6 (10%)	17 (5.7%)
Joint stiffness	2 (3.3%)	4 (1.3%)
Joint swelling	2 (3.3%)	3 (1%)
Lameness	1 (1.7%)	10 (3.3%)
Metritis	3 (5%)	6 (2%)
Vaginal hemorrhage	0 (0%)	1 (0.3%)
Abortion	1 (1.7%)	2 (0.7%)
Rectal prolapse	1 (1.7%)	2 (0.7%)
Dystocia	1 (1.7%)	4 (1.3%)
Found dead	0 (0%)	3 (1%)
Vaginal prolapse	0 (0%)	2 (0.7%)
Mammary gland edema	1 (1.7%)	0 (0%)
Mastitis	1 (1.7%)	0 (0%)

**Table 9:** Frequency distribution of sow outcome by treatment; number and percentages in brackets.

	Control (T01)	Vaccinated (T02 + T03)
Total Number (percentage)	60 (100%)	299 (100%)
Farrowed	57 (95%)	286 (95.7%)
Abortion	1 (1.7%)	2 (0.7%)
Not pregnant	2 (3.3%)	8 (2.7%)
Culled/dead	0 (0%)	3 (1%)

**Table 10:** Back-transformed mean % of piglets per litter and treatment.

	Control (T01)	Vaccinated (T02 + T03)
Number of sows that farrowed/total (%)	57/60 (95.0%)	286/299 (95.6%)
Born Alive	87.1%	90.5%
Born Normal	82%	86.5%
Low viability	1.1%	1.1%
Stillborn	10.5%	7.1%
Mummies	0.4%	0.7%

## Data Availability

The data presented in this study are available upon reasonable request from the corresponding author.
